# Low Levels of Sex Hormone-Binding Globulin Constitute an Independent Risk Factor for Arterial Stiffness in Korean Women

**DOI:** 10.1155/2017/6956495

**Published:** 2017-10-30

**Authors:** Kunhee Han, Hyejin Chun, Moon-Jong Kim, Doo-Yeoun Cho, Soo-Hyun Lee, Bo Youn Won, Kwang-Min Kim, Nam-Seok Joo, Young-Sang Kim

**Affiliations:** ^1^Department of Family Medicine, CHA Bundang Medical Centre, CHA University, Seongnam 13496, Republic of Korea; ^2^Department of Clinical Pharmacology, CHA Bundang Medical Centre, CHA University, Seongnam 13496, Republic of Korea; ^3^Department of Family Medicine and Community Health, Ajou University, Suwon 16499, Republic of Korea

## Abstract

The association between sex hormone-binding globulin (SHBG) and arterial stiffness in women is not conclusive. In addition, obesity might also be involved in the relationship between SHBG and atherosclerosis. The aim of this study was to determine the relationship between SHBG and arterial stiffness in association with central obesity in women. This cross-sectional study included 381 women who participated in the health checkup programs in one hospital. The brachial-ankle pulse wave velocity (baPWV) was measured as a marker for arterial stiffness. A negative correlation was observed between SHBG levels and baPWV (rho = −0.281). The relationship was significant even after adjusting for potential confounders (beta = −0.087 in fully adjusted model). After considering the interaction between central obesity and SHBG levels, the significant association was evident only in obese women (*P* for interaction = 0.025). Adjustment for a 10-year atherosclerotic cardiovascular disease (ASCVD) risk scores, instead of each cardiovascular risk factor individually, did not affect the significance of the relationship between SHBG levels and baPWV. Serum levels of SHBG were negatively associated with arterial stiffness independent of cardiovascular risk factors or 10-year ASCVD risk scores in Korean women. The relationship may be potentiated by central obesity.

## 1. Introduction

Sex hormone-binding globulin (SHBG) is a glycoprotein that binds to sex hormones. Although the affinity of androgen for SHBG is stronger than that of estrogen [1], SHBG regulates the bioavailability of both hormones. In addition, it is clear that the cell membranes of selected tissues contain a receptor for SHBG [2]. Hence, SHBG may have both a direct and an indirect effect on metabolism in the human body. Several clinical studies have demonstrated the metabolic function of SHBG. Low levels of serum SHBG predicted metabolic syndrome and type 2 diabetes in men and women [3–6]. Furthermore, SHBG levels have been found to be associated with cardiovascular disease (CVD) in men [7].

Low levels of SHBG have also been linked with indices for atherosclerosis such as coronary artery calcification or carotid artery intima-media thickness in women [8, 9]. Arterial stiffness is also recognized as a good surrogate marker for subclinical atherosclerosis [10]. Arterial stiffness is influenced by age, hypertension, diabetes, atherosclerosis, and chronic kidney disease [11]. To date, only a few studies have investigated the association between SHBG and arterial stiffness [12–14]. Two of the studies have shown a weak correlation before adjustment for potential confounders [12, 14].

Although central obesity is considered an important component of metabolic syndrome, it was not included in cardiovascular risk estimation models such as Framingham risk and atherosclerotic cardiovascular disease (ASCVD) risk calculator [15]. Meanwhile, obesity has been thought to be a major determinant of circulating SHBG levels [16]. Therefore, obesity might also be involved in the relationship between SHBG and atherosclerosis.

Observational studies have reported a relationship between SHBG levels and the incidence of cardiovascular events in women [17–19]. However, these results were not consistent. Only a few studies have investigated the association between SHBG levels and atherosclerosis in women [8, 9]. In these studies, influence of obesity on the relationship between SHBG and atherosclerosis has also been underestimated. Therefore, in the present study, we investigated the relationship between SHBG and brachial-ankle pulse wave velocity (baPWV), a surrogate marker for arterial stiffness, in women. In addition, we attempted to determine whether this relationship was dependent on central obesity.

## 2. Study Population and Methods

### 2.1. Study Design and Participants

In this cross-sectional study, women who visited CHA Bundang Medical Center for health checkups from 2006 to 2007 were enrolled. All participants voluntarily took part in our study without any reward. Informed consent was obtained from all participants. The total number of women with data on SHBG levels and baPWV was 459. Amongst them, participants receiving hormone therapy or steroid treatment, those diagnosed with acute disease, abnormal liver, kidney, or thyroid function and those with a history of stroke, angina, myocardial infarction, or cancer were excluded. Finally, 381 women were analyzed in the current study. This study was approved by the institutional review board of CHA Hospital in Korea.

### 2.2. Measurements

The information on the medical history and lifestyle habits was collected using self-report questionnaires. Participants with smoking history were categorized into nonsmokers, ex-smokers, and current smokers. Participants with drinking history were categorized into current consumers and nonconsumers. Exercise was classified into routine (moderate-to-strenuous intensity, 3 times a week or more frequent) and nonroutine. Menopause was defined as the absence of menstruation during the last year and follicle-stimulating hormone (FSH) > 40 mIU/mL.

Height and weight were measured in a standing position without shoes and recorded to the first decimal point in centimeters and kilograms, respectively. BMI was calculated as the weight in kilograms divided by the square of the height in meters. Waist circumference was measured midway between the lower rib margin and the iliac crest in a standing position. Central obesity was defined as a waist circumference of 80 cm or greater [20]. Blood pressure (BP) was measured after resting for 10 min in a sitting position using an automatic sphygmomanometer. Mean BP was defined as a mean of systolic and diastolic BPs of each individual.

### 2.3. Blood Sampling

Fasting blood samples were drawn from the antecubital area in the morning. Serum samples were stored at 4°C and analyzed within a day of sampling. Glucose, creatinine, liver enzymes, and lipid profiles were examined using an automatic analyzer (Hitachi 7600; Hitachi, Tokyo, Japan). The glomerular filtration rate (GFR) was estimated using the modification of diet in renal disease method [21]. Serum concentrations of SHBG were analyzed by electrochemiluminescent immunoassay (Roche Diagnostics, Mannheim, Germany) with intra- and interassay coefficients of variation of ≤4% and ≤6%, respectively. Serum concentrations of FSH were analyzed by chemiluminescent immunoassay (ADVIA Centaur; Siemens Diagnostics, Tarrytown, NY, USA).

### 2.4. Arterial Stiffness and ASCVD Risk Score

The baPWV was measured using the Colin VP-1000 Plus system (Omron, Kyoto, Japan). Patients were examined in a supine position, with a volume plethysmographic sensor in cuffs on both the brachia and ankles. After 15 minutes of rest, the participant's volume pulse form was recorded, and the time intervals between the wavefront of the arm and that of the ankles were calculated. The distance between the arm and leg points was calculated automatically.

The 10-year ASCVD risk score was calculated by the method introduced in the American College of Cardiology (ACC) and the American Heart Association (AHA) guideline in 2013 [15]. The variables of this calculation included sex, age, race, systolic blood pressure, serum concentration of total and high-density lipoprotein (HDL) cholesterol, presence of diabetes mellitus, hypertension treatment, and smoking status.

### 2.5. Statistical Analyses

All analyses were conducted using SPSS statistical analysis software, version 24.0 (IBM, Armonk, NY, USA). For descriptive analysis, the results were expressed as median (interquartile range) or number (proportion). The variables were compared using Mann–Whitney *U* test or chi-squared test according to central obesity. The correlation between SHBG and cardiovascular risk factors was analyzed using Spearman's rank correlation test. The variables of SHBG, glucose, waist circumference, and mean BP were logarithmically transformed to reduce skewness of distribution in the parametric statistical analyses. The multivariate regression models were formed to assess the influence of SHBG levels on baPWV. Model 1 included the variables of age, waist circumference, and mean BP; model 2 additionally adjusted for the variables of glucose, FSH, hypertension, diabetes, smoking, drinking, and exercise. Model 3 included the variables of ASCVD risk score and FSH.

To control for the influence of central obesity, the association between SHBG levels and PWV was reassessed using the linear regression model including the interaction term between SHBG levels and central obesity. Similarly, central obesity and the interaction term were included in the multivariate regression models.

## 3. Results

The median age of the subjects was 53 years. Of the 381 subjects, 240 women were obese (62.9%), whereas the remaining 141 were not (37.0%). Most of the metabolic parameters were significantly different between obese and nonobese subjects. The median SHBG levels in nonobese and obese subjects were 83.8 and 61.4 nmol/L, respectively. Although the 10-year ASCVD risk was low in both groups (0.9 and 2.2% in the nonobese and obese group, resp.), this difference was statistically significant (*P* < 0.001; Table 1).

The association of SHBG levels with the cardiovascular risk factors is shown in Table 2. The correlation coefficient between SHBG levels and baPWV was −0.281 (*P* < 0.001). The baPWV was positively correlated with most of the metabolic parameters. In contrast, the correlation between SHBG level and most of the parameters was negative. The correlation of HDL cholesterol with PWV and SHBG levels was negative and positive, respectively.

The association of SHBG levels with PWV was further analyzed after adjusting for various cardiovascular risk factors (Table 3). After controlling for age, waist circumference, and BP, SHBG levels were found to be negatively correlated with PWV (model 1). Additional adjustment for potential confounders did not change the significance of the negative association (model 2). However, against the expectation from the correlation analyses, the regression coefficients of waist circumference were −0.093 and −0.113 in model 1 (*P* = 0.020) and model 2 (*P* = 0.004), respectively. When ASCVD risk score was included in the regression model instead of each cardiovascular risk factor individually (model 3), the relationship of SHBG levels with PWV was also significant (beta = −0.082; *P* = 0.037). Furthermore, FSH levels were not related to PWV in the multivariate regression models (model 2 and model 3).

**Figure 1:**
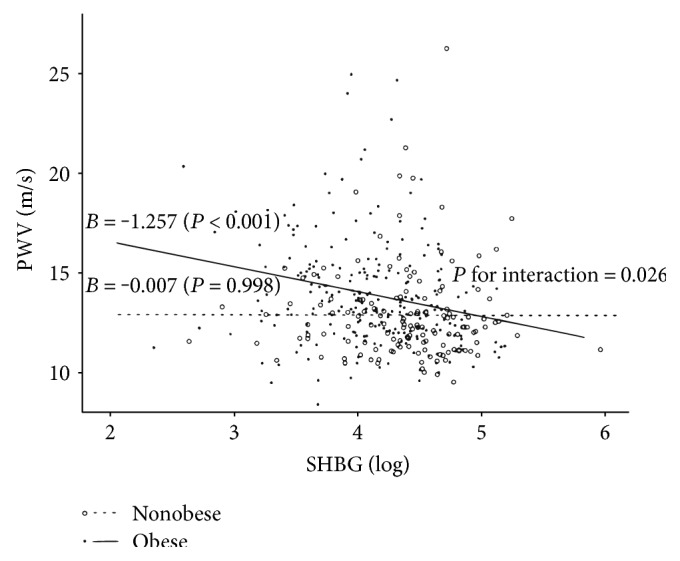
Scatter plot and regression lines between sex hormone-binding globulin and brachial-ankle pulse wave velocity according to central obesity.

**Figure 2:**
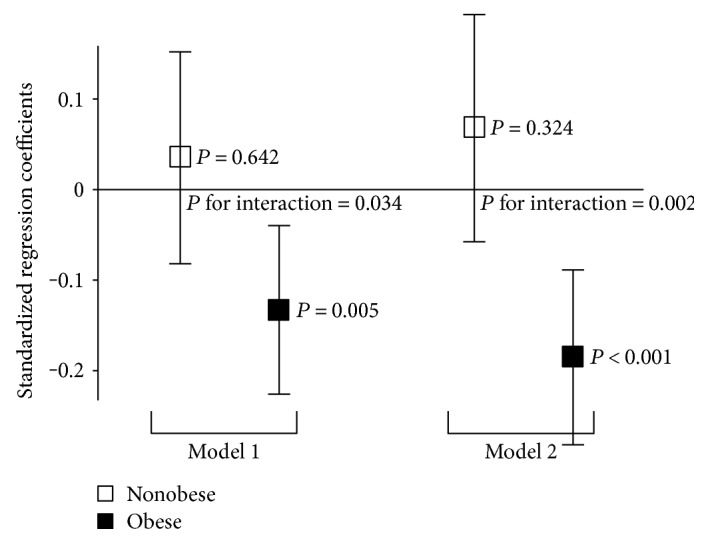
Standardized regression coefficients of sex hormone-binding globulin for brachial-ankle pulse wave velocity according to central obesity. Error bars represent standard errors of mean. Model 2 includes the variables of SHBG, central obesity, their interaction, and potential confounders (age, mean BP, glucose, FSH, hypertension, DM, smoking, drinking, and exercise); model 3 includes the 10-year ASCVD risk score instead of cardiovascular risk factors in model 1.

After considering the interaction term between SHBG levels and central obesity, SHBG levels were found to be negatively correlated with PWV only in the obese group; the interaction was significant (*P* for interaction = 0.025; Figure 1). The standardized regression coefficients of SHBG levels for PWV, after adjusting for potential confounders considered in the regression models (Table 3), are shown in Figure 2. The association was significant only for the obese group in the multivariate models. The interaction term was also significant in both models.

## 4. Discussion

In the present study, SHBG levels were found to be negatively correlated with baPWV in Korean women. The relationship was persistently significant even after adjusting for various cardiovascular risk factors or the 10-year ASCVD risk score. When interaction terms between central obesity and SHBG levels were considered, significant correlation was observed only for the centrally obese group.

Pulse wave velocity (PWV) is a method of measuring arterial stiffness that indirectly presents as a surrogate marker for CVD risk and is closely correlated with the parameters of metabolic syndrome [22, 23]. Carotid-femoral PWV (cfPWV) is considered the gold-standard measurement for arterial stiffness [24]. Compared to cfPWV, baPWV exhibits a similar extent of associations with CVD risk factors and clinical events [25]. baPWV has recently become a popular technique for screening vascular damages in large populations because of its simple and noninvasive procedure [26]. For these reasons, our study showed that lower levels of SHBG constitute a risk factor for subclinical atherosclerosis, especially in obese women. The association between SHBG levels and the indices of arterial stiffness, including baPWV, was also investigated in a few prior studies [12–14]. However, the significant relationship was demonstrated only before the adjustment for potential confounders.

Similar to our study, various studies have determined that low levels of serum SHBG constitute a risk factor for atherosclerosis and CVD in women [8, 9, 27]. Because SHBG regulates the levels of sex hormone, our results may be influenced by estrogen or androgen. However, a study reported that only a few CVD risk factors were correlated with serum concentrations of estradiol and testosterone, while almost all of the risk factors were correlated with free androgen index; the denominator of which is SHBG level [28]. Similarly, total testosterone and total estradiol were not found to be related to cardiovascular risk factors in another study for premenopausal women [29]. Although our study did not measure the concentrations of serum sex hormones, according to multivariate analyses, the FSH levels were not significant factors. The menopause status was also not a significant confounder (data not shown). In our study population, we hypothesized that arterial stiffness was influenced not by changes in sex hormone levels but by aging itself. Based upon a previous review [30], insulin may mediate the association between SHBG production and atherosclerosis. Insulin resistance is an important mechanism to explain progression of atherosclerosis. In addition, several reports showed an inverse relationship between serum insulin and SHBG levels [31, 32]. However, according to recent studies, insulin may not regulate the production of SHBG [33, 34]. SHBG production is regulated by several inflammatory cytokines and adiponectin [35]. Further studies should examine the effects of the related hormones and cytokines.

The relationship between SHBG levels and CV risk factors may differ depending on sex. In men, although high levels of SHBG were associated with favorable BP and lipid profiles independent of testosterone [36], SHBG levels were not related to atherosclerosis [27, 37]. Instead, low testosterone levels were a risk factor for atherosclerosis [38–41] as well as CVD mortality [42–44]. These findings suggest that testosterone, rather than SHBG, may influence atherosclerosis and CVD. Contrary to the findings in men, several studies have shown a significant relationship between SHBG levels and subclinical atherosclerosis in women [8, 9, 27]. Age-related increase in serum concentration of SHBG is universal in men. In contrast, age-related decline of SHBG levels has been previously described in women [19]; this finding was also observed in our study. Among the factors that regulate SHBG in the human body, insulin plays a major role in women, whereas a decrease in insulin-like growth factor 1 is the predominant regulating factor in men [45].

Central obesity has been thought to be a major determinant of SHBG levels [16]. Thus, central obesity may be a confounding factor for the relationship between SHBG levels and cardiovascular risk. In an observational study, the relationship between SHBG levels and incidental cardiovascular events was not found to be independent of obesity and other cardiovascular risk factors [18]. In our multivariate analyses, baPWV was negatively correlated with waist circumference; this finding was a complete opposite of the result of simple correlation analyses. It suggests that central obesity is not a direct risk factor for arterial stiffness. Additionally in the ASCVD risk calculator [15], the factor of obesity is not included in the equation. For these reasons, it was hypothesized that stratifying subjects by their obesity status may influence the association between SHBG levels and arterial stiffness. In nonobese women, this association was not statistically significant. Specifically, low levels of serum SHBG was a risk factor for arterial stiffness especially in obese women.

The 10-year ASCVD risk score was developed based on highly reliable studies [15]. Although it has not been sufficiently demonstrated whether this tool fits well for the Korean population [46], its usefulness is widely accepted in clinical settings. In the present study, ASCVD risk score was found to be strongly correlated with arterial stiffness estimated by PWV. In addition, adjusting for this score instead of each variable related to CVD did not affect the relationship between SHBG levels and PWV.

Our study had several limitations. First, as the study design was cross-sectional, causal relationships were not confirmed. Second, we did not measure additional markers for atherosclerosis. Direct visualization of the blood vessels may facilitate the assessment of the degree of atherosclerosis. Although baPWV is an indirect marker for atherosclerosis, it predicted CVD events and mortality [47]. Third, data on serum concentrations of hormones such as estradiol, testosterone, and insulin were not assayed. According to prior studies [48, 49], the influence of sex hormones on CVD risk factors was not obvious. Contrary to most of the previous studies, our study included premenopausal women; the variables of either menopause status or FSH levels were not significant in the multivariate models including the age variable. Although these factors influence sex hormone levels, they may not be a risk factor for arterial stiffness independent of age. As mentioned before, insulin plays a strong role in the regulation of CVD risk and SHBG metabolism. Nonetheless, further measurements of additional hormonal factors are necessary in subsequent studies. Fourth, although our study showed the interaction by obesity, the information of polycystic ovary syndrome was not collected.

In conclusion, serum levels of SHBG were negatively correlated with baPWV, independent of conventional cardiovascular risk factors or 10-year ASCVD risk scores in Korean women. Although the association was not influenced by the menopause status, central obesity potentiated the association. Further longitudinal studies including additional variables may aid in the understanding of the effect of SHBG on subclinical atherosclerosis.

## Figures and Tables

**Table 1:** General characteristics of the subjects according to central obesity.

	Total (*N* = 381)	Nonobese (*N* = 141)	Obese (*N* = 240)	*P*
Age	53.0 (47.0–58.0)	50.0 (45.0–55.5)	54.0 (48.0–59.0)	<0.001
Menopause	226 (59.3%)	69 (48.9%)	157 (65.4%)	0.002
Alcohol consumer	157 (41.2%)	53 (37.6%)	104 (43.3%)	0.321
Smoking				0.259
Nonsmoker	348 (91.3%)	128 (90.8%)	220 (91.7%)	
Ex-smoker	10 (2.6%)	6 (4.3%)	4 (1.7%)	
Current smoker	23 (6.0%)	7 (5.0%)	16 (6.7%)	
Routine exercise	151 (39.6%)	64 (45.4%)	87 (36.3%)	0.098
Hypertension	45 (11.8%)	8 (5.7%)	37 (15.4%)	0.007
Type 2 diabetes	11 (2.9%)	3 (2.1%)	8 (3.3%)	0.718
Systolic BP (mmHg)	118.0 (109.0–128.0)	112.0 (105.0–122.5)	121.0 (112.0–132.8)	<0.001
Diastolic BP (mmHg)	78.0 (71.0–85.0)	73.0 (67.0–80.5)	80.0 (73.0–86.0)	<0.001
BMI (kg/m2)	23.5 (21.5–26.1)	21.0 (20.1–22.0)	25.0 (23.5–27.0)	<0.001
Waist circumference (cm)	82.7 (77.3–88.5)	75.4 (73.2–77.8)	86.4 (83.2–90.5)	<0.001
Glucose (mg/dL)	93.0 (87.0–100.0)	92.0 (84.5–97.0)	93.0 (88.0–101.0)	0.005
Total cholesterol (mg/dL)	195.0 (173.0–219.0)	187.0 (165.0–207.5)	199.0 (177.3–222.0)	<0.001
Triglyceride (mg/dL)	98.0 (70.0–137.5)	82.0 (60.0–111.5)	108.5 (74.0–147.8)	<0.001
HDL cholesterol (mg/dL)	53.3 (45.4–61.7)	56.9 (47.7–66.2)	51.0 (44.7–58.9)	<0.001
AST (U/L)	21.0 (18.0–25.0)	21.0 (18.0–26.0)	21.0 (18.0–24.0)	0.614
ALT (U/L)	18.0 (14.0–24.0)	16.0 (13.0–21.0)	18.0 (15.0–24.0)	0.003
Estimated GFR (mL/min/1.73m^2^)	77.2 (69.2–82.1)	77.8 (69.6–82.4)	77.0 (69.1–82.1)	0.458
baPWV (m/s)	13.0 (11.9–14.8)	12.3 (11.4–13.7)	13.3 (12.1–15.1)	<0.001
SHBG (nmol/L)	69.9 (49.0–94.9)	83.8 (61.0–111.6)	61.4 (43.4–87.9)	<0.001
FSH (mIU/mL)	41.7 (7.3–63.2)	33.3 (5.8–60.1)	45.5 (9.2–63.3)	0.166
Ten-year ASCVD risk (%)	1.6 (0.7–3.5)	0.9 (0.5–2.0)	2.2 (1.0–4.2)	<0.001

Data are expressed as median (interquartile range) or number (proportion). *P* values are derived using Mann–Whitney *U* test or chi-squared test; BP: blood pressure; BMI: body mass index; HDL: high-density lipoprotein; ALT: alanine aminotransferase; AST: aspartate aminotransferase; GFR: glomerular filtration rate; baPWV: brachial-ankle pulse wave velocity; SHBG: sex hormone-binding globulin; FSH: follicle-stimulating hormone; ASCVD: atherosclerotic cardiovascular disease.

**Table 2:** Correlation of brachial-ankle pulse wave velocity and sex hormone-binding globulin with metabolic parameters.

	PWV		SHBG	
	rho	*P*	rho	*P*
baPWV			−0.281	<0.001
Ten-year ASCVD risk	0.713	<0.001	−0.234	<0.001
Age	0.608	<0.001	−0.145	0.005
Systolic BP	0.666	<0.001	−0.231	<0.001
Diastolic BP	0.593	<0.001	−0.189	<0.001
BMI	0.202	<0.001	−0.325	<0.001
Waist circumference	0.280	<0.001	−0.364	<0.001
Glucose	0.351	<0.001	−0.218	<0.001
Total cholesterol	0.281	<0.001	−0.183	<0.001
Triglyceride	0.345	<0.001	−0.325	<0.001
HDL cholesterol	−0.199	<0.001	0.311	<0.001
FSH	0.414	<0.001	0.381	<0.001

The rho represents the Spearman correlation coefficient; baPWV: brachial-ankle pulse wave velocity; SHBG: sex hormone-binding globulin; ASCVD: atherosclerotic cardiovascular disease; BP: blood pressure; BMI: body mass index; HDL: high-density lipoprotein; FSH: follicle-stimulating hormone.

**Table 3:** Multivariate regression models of sex hormone-binding globulin for brachial-ankle pulse wave velocity.

	Model 1		Model 2		Model 3	
	Beta (SE)	*P*	Beta (SE)	*P*	Beta (SE)	*P*
SHBG	−0.098 (0.039)	0.012	−0.087 (0.038)	0.023	−0.082 (0.039)	0.037
Age	0.424 (0.040)	<0.001	0.376 (0.048)	<0.001		
Waist circumference	−0.093 (0.040)	0.020	−0.113 (0.039)	0.004		
Mean BP	0.424 (0.041)	<0.001	0.397 (0.039)	<0.001		
Glucose			0.094 (0.040)	0.020		
Ten-year ASCVD risk					0.642 (0.045)	<0.001
FSH			0.030 (0.043)	0.048	0.035 (0.045)	0.414
Adjusted *R* square	0.516		0.566		0.463	

Beta represents standardized regression coefficient. Model 1 includes the variables of SHBG, age, waist circumference, and mean BP; model 2 additionally includes the variables of glucose, FSH, menopause state, hypertension, DM, smoking, drinking, and exercise; model 3 includes the variables of SHBG, ASCVD risk, and FSH; SHBG: sex hormone-binding globulin; BP: blood pressure; ASCVD: atherosclerotic cardiovascular disease; FSH: follicle-stimulating hormone.
